# The influence of bone marrow edema for the assessment of the boundaries of necrotic lesions in patients with osteonecrosis of the femoral head

**DOI:** 10.1038/s41598-022-23427-y

**Published:** 2022-11-04

**Authors:** Satoshi Ikemura, Goro Motomura, Ryosuke Yamaguchi, Takeshi Utsunomiya, Satoshi Hamai, Masanori Fujii, Shinya kawahara, Taishi Sato, Daisuke Hara, Kyohei shiomoto, Takuaki Yamamoto, Yasuharu Nakashima

**Affiliations:** 1 Department of Orthopaedic Surgery, Graduate School of Medical Sciences, Kyushu University, 3-1-1 Maidashi, Higashi-Ku, Fukuoka, 812-8582 Japan; 2 Department of Orthopaedic Surgery, Faculty of Medicine, Fukuoka University, 7-45-1 Nanakuma, 12 Jonan-Ku, Fukuoka, 814-0133 Japan

**Keywords:** Anatomy, Diseases, Pathogenesis, Rheumatology

## Abstract

This study aimed to investigate the influence of bone marrow edema (BME) for the assessment of the boundaries of necrotic lesions using unenhanced and contrast-enhanced (CE) magnetic resonance (MR) images in patients with osteonecrosis of the femoral head (ONFH). We retrospectively reviewed 72 consecutive hips in 55 patients of ONFH that were Association Research Circulation Osseous (ARCO) stage III or higher and underwent both unenhanced and contrast-enhanced MR imaging between January 2005 and February 2016. The degree of extension of BMEs, and the boundaries of the necrotic lesions were compared using unenhanced and CE MR images on both mid coronal and mid oblique-axial slices. Forty-two percent of the coronal T1 images, 40% of the coronal fat-saturated T2 images, and 48% of the oblique-axial T1 images showed differences in the boundaries of necrotic lesion, by comparison with those of CET1-weighted MR images. The boundaries of necrotic lesions were clearly detected in all hips on CE coronal slices and 97% of all hips on CE oblique-axial slices. The BME grade in the difference group was significantly higher than in the non-difference group on the coronal plane (*P* = 0.0058). There were significant differences between the BME grade and duration from the onset of hip pain to MR imaging examination. Multivariate analyses revealed that the duration from the onset to MR imaging examination in both coronal (*P* = 0.0008) and oblique-axial slices (*P* = 0.0143) were independently associated with differences in the boundary of necrotic lesion between T1 and CET1-weighted MR images. Our findings suggest that unenhanced MR image may be insufficient for a precise assessment of the boundaries of the necrotic lesions for ONFH cases in the early phase of subchondral collapse due to the diffuse BME.

## Introduction

Osteonecrosis of the femoral head (ONFH) is characterized by substantial risk of femoral head collapse which usually leads to secondary osteoarthritis of the hip joint with severe hip pain and joint dysfunction^1–4^. Precise evaluation of the boundary of a necrotic lesion is necessary, since it is considered that the location of the necrotic lesion is an important factor for predicting further collapse as well as size of the necrotic lesion^1,5–9^. Both clinical and histological studies have highlighted the importance of the lateral boundary of the necrotic lesion on occurrence of a collapse^1,10,11^. Additionally, a recent study indicated that location of the anterior boundary of a necrotic lesion also plays an important role in the occurrence of a collapse^12^.

Surgical treatment is necessary when subchondral collapse occurs in patients with ONFH to relieve pain and to obtain good hip function. Transtrochanteric rotational osteotomy and transtrochanteric curved varus osteotomy are joint-preserving procedures used to treat ONFH^13–15^. Preoperative precise evaluation of the necrotic area at the articular surface is necessary for preoperative planning for osteotomy, since the postoperative intact ratio (intact area of the femoral head/weight-bearing area of the acetabulum) is a critical factor for influencing the clinical result after the osteotomy^13–15^.

The T1 low-intensity band on magnetic resonance imaging (MRI) is generally used to evaluate the necrotic area^16,17^. However, Sakai et al. reported that the rate of detection of the boundaries of necrotic lesion in collapsed ONFH cases (the Association Research Circulation Osseous [ARCO] stage III or IV) was lower than that in non-collapsed ONFH cases (ARCO stage I or II), based on unenhanced T1-weighted MR imaging^18^. Although a previous study reported that contrast-enhanced (CE) MR imaging was useful for the detection of the necrotic boundary even in collapsed ONFH cases, little is known concerning the factors that influence the assessment of the boundaries of necrotic lesions^18^. We hypothesized that the bone marrow edema (BME), which is considered to result in secondary to subchondral collapse on MR images, would affect the assessment of boundaries of necrotic lesions. This study aimed to compare the boundaries of the necrotic lesions between unenhanced and CE MR imaging on both coronal and oblique-axial planes, and to determine the factors influencing the difference in their appearances in ONFH patients with subchondral collapse.

## Materials and methods

**Figure 1:**
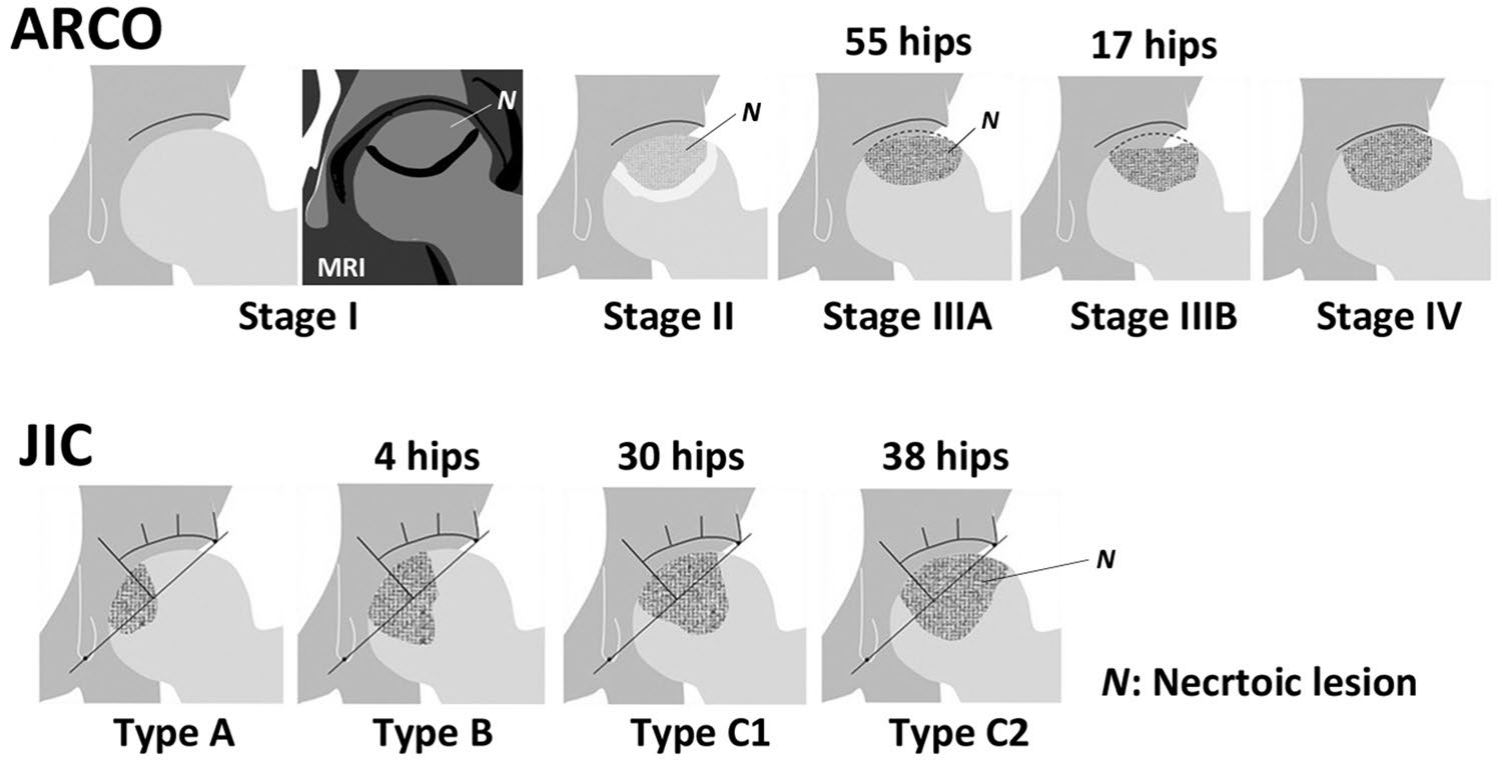
Diagrams showing the 2019 revised Association Research Circulation Osseous (ARCO) and the Japanese Investigation Committee (JIC) systems. ARCO Stage I, T1 low-intensity band on MR imaging without any specific findings on plain radiographs; Stage II, demarcating sclerosis without collapse on plain radiographs; Stage IIIA, lesser than or equal to 2 mm collapse; stage IIIB, greater than 2 mm collapse; Stage IV, osteoarthritic changes. In this study, 55 hips are classified as stage IIIA and 17 hips as stage IIIB. JIC Type A, necrotic area occupies the medial one-third or less of the weight-bearing portion; Type B, medial two-thirds or less; Type C1, more than two-thirds but not extending to the acetabular rim; Type C2, more than two-thirds and extending to the acetabular rim. In this study, 55 hips are classified as stage IIIA and 17 hips as stage IIIB, and 4 hips are type B, 30 hips are type C1 and 38 hips are type C2.

This study was approved by our institutional review board. It was conducted in accordance with the Declaration of Helsinki. Written informed consent was obtained from all patients prior to study. MRI was performed on 828 hips in 588 patients with ONFH between January 2005 and February 2016. Among them, we retrospectively reviewed 72 consecutive hips in 55 patients who underwent a CE MR imaging examination for the reason that it was difficult to assess the boundary of necrotic lesion due to BME after the subchondral collapse (ARCO stage III or higher, Fig. 1) or it was difficult to differentiate ONFH from subchondral insufficiency fracture of the femoral head or transient osteoporosis of the hip, using unenhanced MRI^19^.

Thirty-two hips were treated with transtrochanteric anterior rotational osteotomy, 2 hips with transtrochanteric posterior rotational osteotomy, 10 hips with transtrochanteric curved varus osteotomy, and 26 hips with prosthetic replacement. Two hips were continuously observed without surgical treatment. Three of 44 (6.8%) cases treated with joint-preserving procedures underwent prosthetic replacement due to osteoarthritic change after the osteotomy (2, 4, and 4 years, respectively). One case (2.2%) underwent prosthetic replacement due to nonunion at the osteotomy site caused by deep infection after the surgery. The survival rate was 91.0% (endpoint: prosthetic replacement), with a mean of 9.1 years after the osteotomies (range, 5 to 15 years). ONFH stage (2019 revised ARCO classification) and the localization of the affected lesion (Japanese Investigation Committee (JIC) classification) are shown in Fig. 1^16,20^. Demarcating sclerosis was present in 51 hips (70.8%) on the anteroposterior (AP) or lateral radiograph^21^.

**Figure 2:**
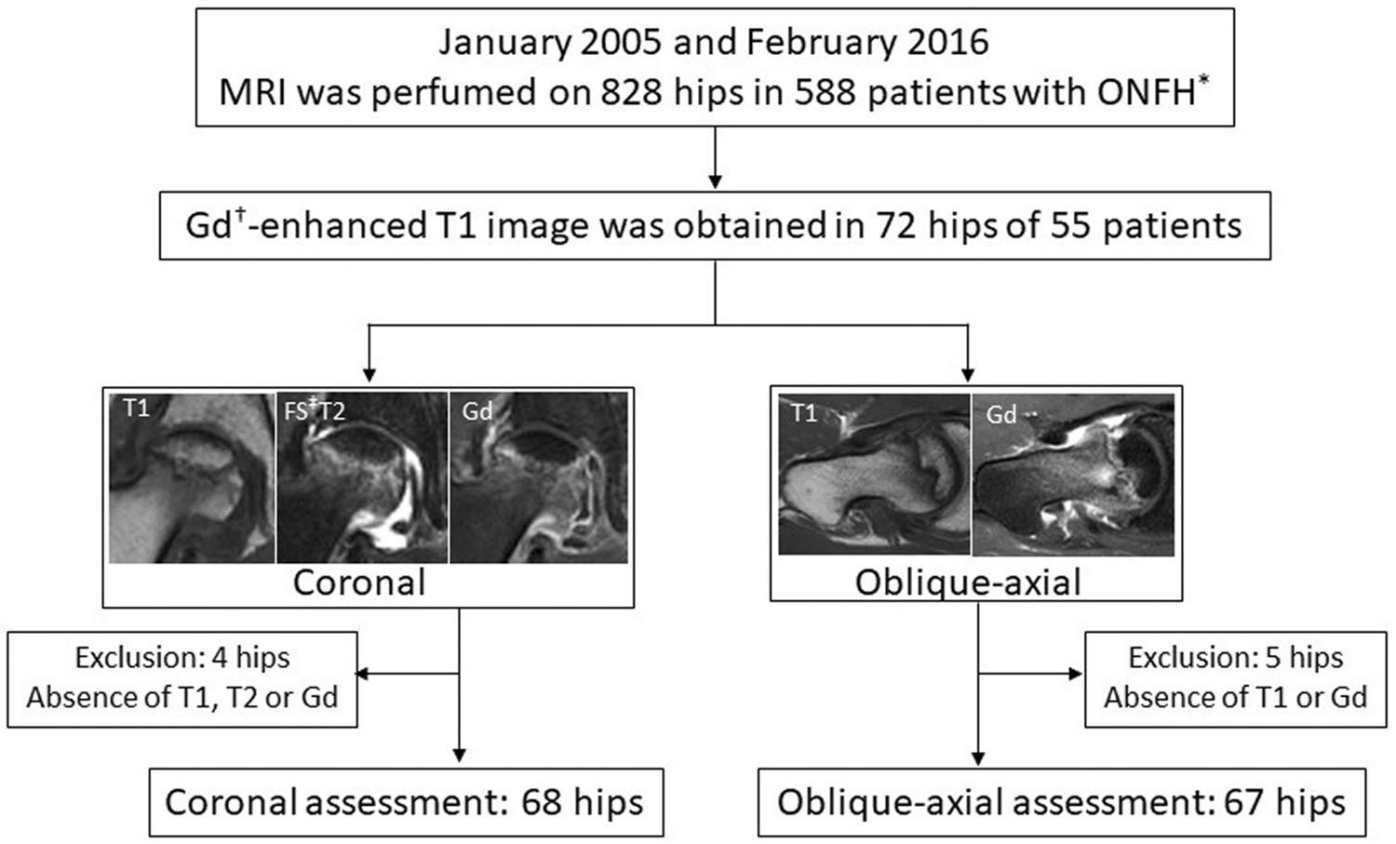
Flowchart demonstrating selection of images of coronal and oblique-axial planes of MRI. *Osteonecrosis of the femoral head, ^†^Gadolinium, ^‡^Fat-saturation.

MR images were obtained with a 1.5-T MR unit (Achieva 1.5 T; Philips Healthcare, Best, The Netherlands) in 52 hips and a 3.0-T MR unit (Achieva 3.0 T; Philips Healthcare, Best, The Netherlands) in 20 hips. After obtaining unenhanced T1- and fat-saturated (FS) T2- (coronal only) weighted spin-echo images (repetition times/echo times [TR/TE] = 400–540/10–18 ms, FST2; 3000–4709/80–100), CET1-weighted images with fat saturation (TR/TE = 620–700/10–18) were obtained by administering 0.2 ml/kg of gadopentetate dimeglumine (Magnevist; Bayer Pharma, Berlin, Germany). All sequences had a 5-mm slice thickness with a 1-mm interslice gap and a field of view of 360 × 360 mm. The duration of the MR imaging examination was 30–40 min. T1-, FST2-, and CET1-weighted images were available in 68 of 72 hips (94.4%) in the coronal plane, and unenhanced and CET1-weighted images were available in 67 of 72 hips (93.1%) in the oblique-axial plane (paralleling the femoral neck axis) (Fig. 2). Based on the medical records, the mean duration from the time of onset of hip pain to MR imaging examination was 4.7 months (range, 0.5–19 months).

**Figure 3:**
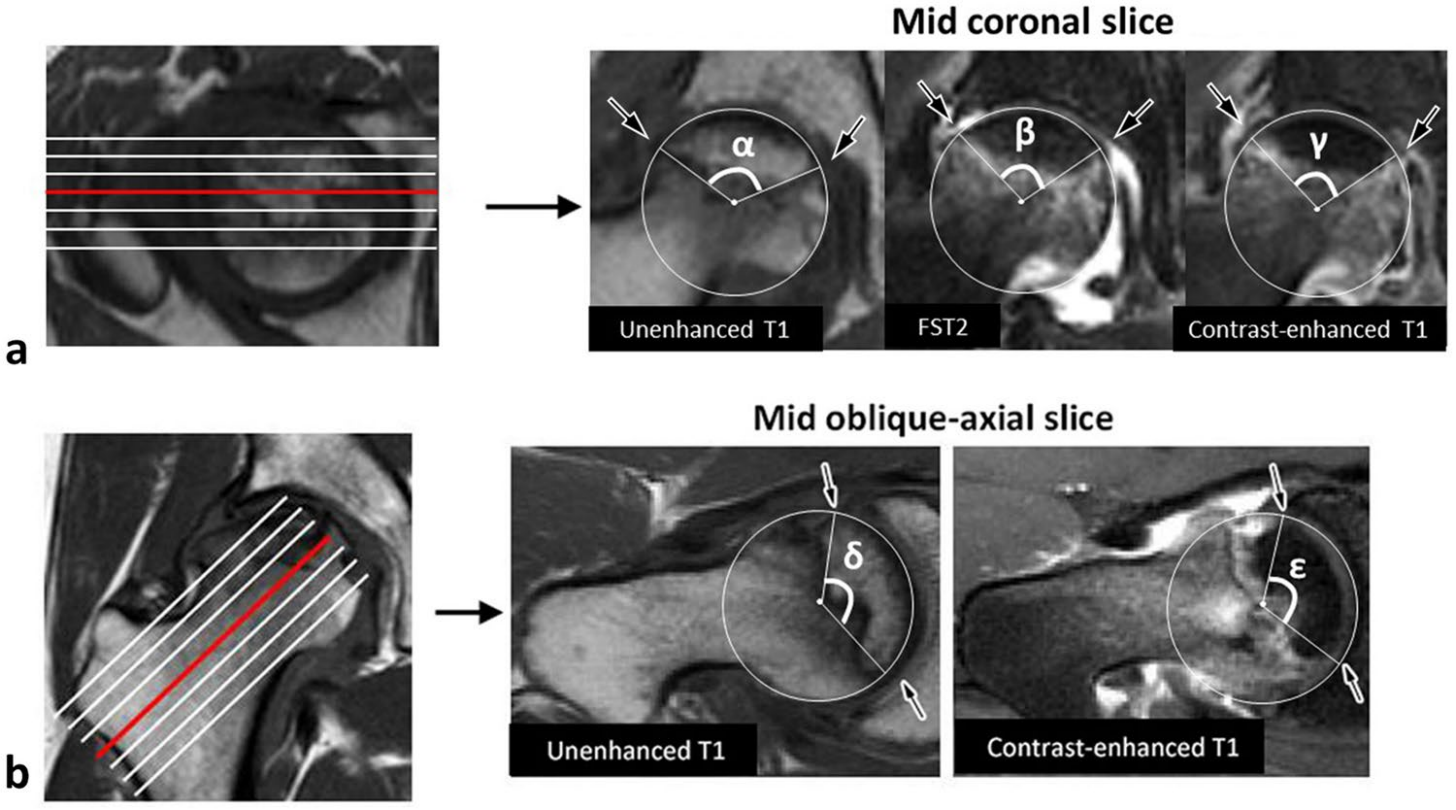
Assessment method for the boundary of the necrotic lesion. (**a**) The medial and lateral boundaries of the necrotic lesion are assessed on both mid coronal unenhanced T1 (*arrows*), fat-saturated (FS) T2 (*arrows*), and contrast-enhanced (CE) T1 images (*arrows*). When the difference with more than 10° between α, β and γ angles is observed, the case is categorized as difference group. In this example, the α, β and γ angles are 118°, 97° and 98°, respectively. Therefore, this case is categorized as difference group in the coronal T1 image and non-difference group in the coronal FST2 image. (**b**) The anterior and posterior boundaries of the necrotic lesion are assessed on both mid oblique-axial unenhanced T1 *(arrows*) and CET1 images (*arrows*). When the difference with more than 10° between δ and ε angles is observed, the case is categorized as difference group. In this example, the γ and δ angles are 129° and 115°, respectively. Therefore, this case is categorized as difference group.

**Figure 4:**
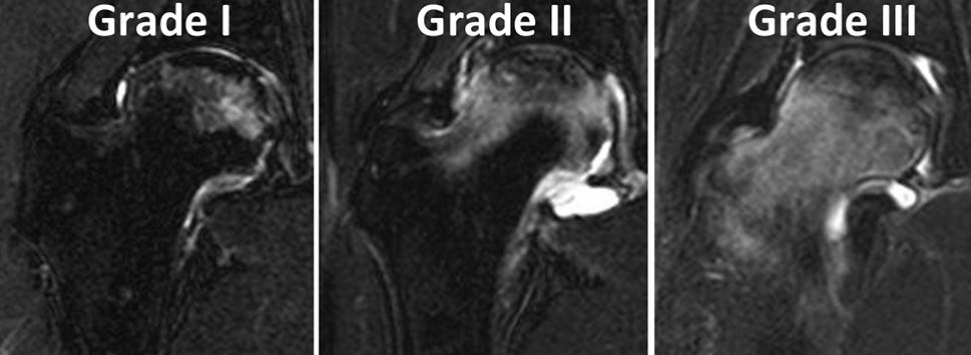
Assessment method for the degree of extension of BME. Grade I; BME is limited within the femoral head. Grade II; BME exceeds the femoral head but remains within the femoral neck. Grade III; BME exceeds the femoral neck reaching the trochanteric area.

In the coronal plane, the boundaries of the necrotic lesion were assessed by mediolateral necrotic angles using T1- and FST2-weighted MR images on mid coronal slice, and were compared with those in the CET1-weighted MR images (Fig. 3). In the oblique axial plane, the boundaries of the necrotic lesion were assessed by AP necrotic angles using T1-weighted MR images on mid oblique-axial slice, and were compared with those in the CET1-weighted MR images (Fig. 3). When the necrotic angles between T1- or FST2-, and CET1-weighed images were different more than 10°, the case was defined as difference group (Fig. 3). The degrees of extension of BMEs (BME grade) were categorized as grade I (within the femoral head), grade II (beyond the femoral head but within the femoral neck), and grade III (beyond the femoral neck) using all slices of T1, FST2, and CET1 images on both coronal- and oblique axial planes (Fig. 4). The assessments were made by two observers (S.I. and T.U.), who are orthopedic surgeons and have extensive experience with diagnostic imaging. To evaluate the intra- and interobserver reproducibility of the assessments of the boundaries between necrotic and living bone comparison between unenhanced and CET1 images, and the assessments of BME grade, the reliabilities of the measurements were evaluated using kappa statistics. A kappa value of 0.21–0.4 indicates a fair agreement; 0.41–0.6, a moderate agreement; and 0.61–0.8, a substantial agreement. A value of > 0.81 is considered to be almost perfect^22^.

After comparison of the boundaries of the necrotic lesions between unenhanced and CE MR images, all hips were divided based on the differences between the two images. Statistical analyses were carried out using the chi-square test or Fisher’s exact probability to compare sex, history of steroid intake or alcohol consumption, stage, type, and presence of radiological demarcating sclerosis between the two groups. The age, BMI, and duration from the onset to MR imaging examination were compared between the two groups using the unpaired *t*-test. The BME grade between the two groups and the relationship between duration from the onset to MR imaging examination and BME grade were analyzed using the Mann–Whitney *U* test and Wilcoxon signed-rank test, respectively. Multivariate analysis was performed to identify parameters associated with differences in the boundaries of necrotic lesions between CE and unenhanced MR images using a stepwise logistic regression with the variable selection (*P* < 0.2). Simple logistic regression analysis was performed to calculate the relationship between the duration from the onset to MR imaging examination and the rate of differences in the boundaries of necrotic lesions between the unenhanced T1- and CET1- weighted MR images. Statistical analyses were performed using JMP Ver. 9.0.1 software (SAS Institute Inc., Cary, NC, USA). *P* values < 0.05 were considered statistically significant.

### Ethics approval and consent to participate

This retrospective study was approved by Kyushu University institutional review board for clinical research (NO. 2019-584). Written informed consent was obtained from all patients prior to study.

## Results

**Figure 5:**
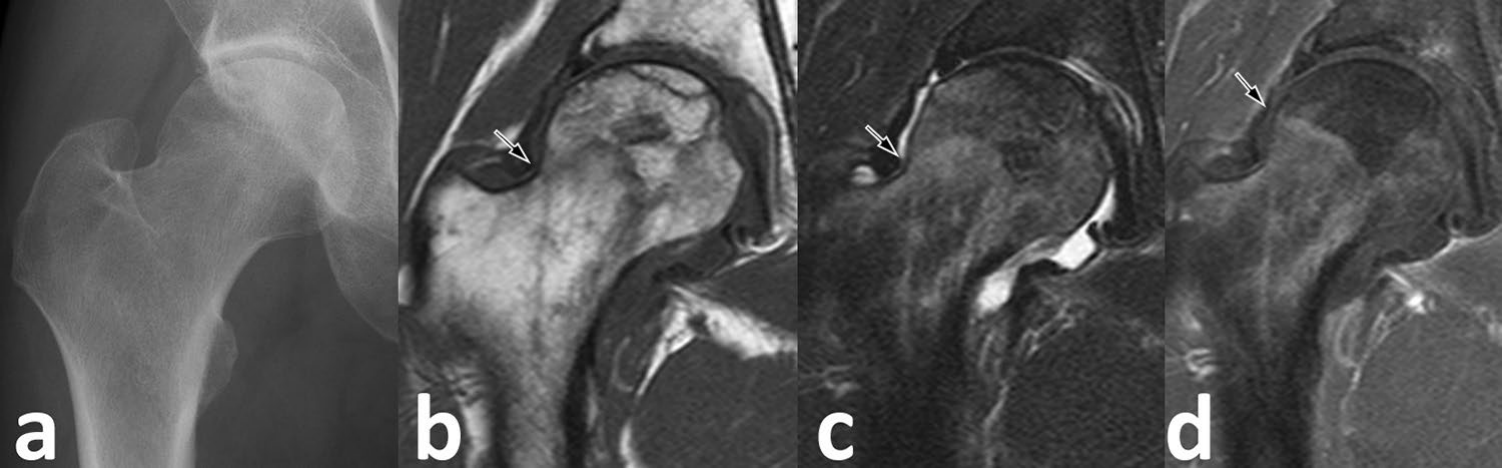
A 43-year-old male with a history of alcohol abuse. (**a**) An anteroposterior (AP) radiograph of the right hip. An obvious collapse is not observed at the femoral head. (**b**,**c**) Diffuse bone marrow edema (BME) secondary to subchondral collapse is observed on unenhanced coronal T1 (**b**) and FST2 (**c**) images (ARCO stage IIIA, BME Grade III (**b**); repetition times/echo times [TR/TE] = 554/10 ms (**c**); TR/TE = 3300/90). The *arrows* indicate the suspected lateral boundary of the necrotic lesion. The necrotic angle is 142° on the T1 and 139° on the FST2 images. (**d**) The contrast-enhanced MR image (TR/TE = 628/10) shows a more medial boundary (*arrow*), and the necrotic angle is 120°.

**Figure 6:**
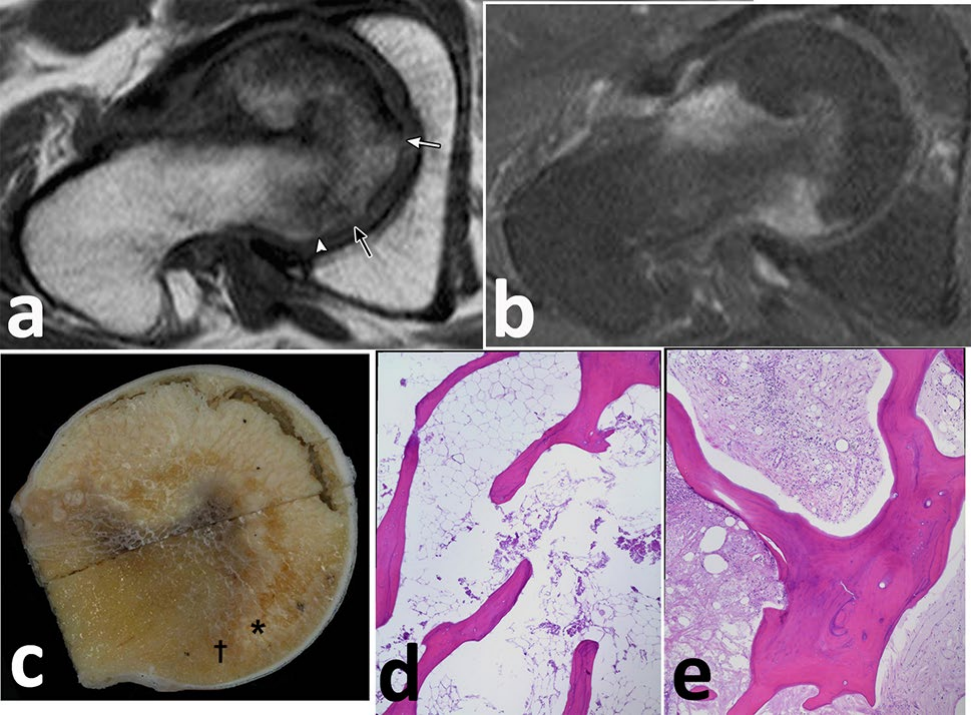
A 52-year-old male with a history of alcohol abuse. (**a**) The posterior boundary of the necrotic lesion on unenhanced oblique-axial T1 image (TR/TE = 500/18 ms) is unclear (*white arrow*, *black arrow*, or *arrowhead*) due to the BME (BME grade II). (**b**) The precise boundary of the necrotic lesion is determined by contrast-enhanced T1 image (TR/TE = 700/18). (**c**) Cut section of the resected femoral head shows yellowish-white necrotic lesion, corresponding to the unenhanced lesion on contrast-enhanced MR image **(b**). (**d**) The histopathological appearance of the necrotic legion (* in **c**), which demonstrates the accumulation of bone marrow cell debris, and the bone trabeculae with empty lacunae beneath the fracture line (haematoxylin and eosin stained; H&E, original magnification 40 ×). (**e**) Repair tissue is found in the viable zone († in **c**), including vascular granulation tissue, fibrous tissue, and appositional bone (H&E, 40 ×).

The boundaries of the necrotic lesions were clearly detected in all 68 hips on the CE coronal slices and in 65 of 67 hips (97%) on the CE oblique-axial slices (Figs. 5 and 6). Twenty-eight hips (42%) of the coronal and 32 hips (48%) of the oblique-axial unenhanced T1-weighted MR images showed the differences in the boundaries of necrotic lesions compared with those of the CET1-weighted MR images. Among them, the boundaries of the necrotic lesions in nine hips (32%) of the mid coronal and in eight hips (25%) of the mid oblique-axial CE MR images were wider than those in the unenhanced T1 images.

**Figure 7:**
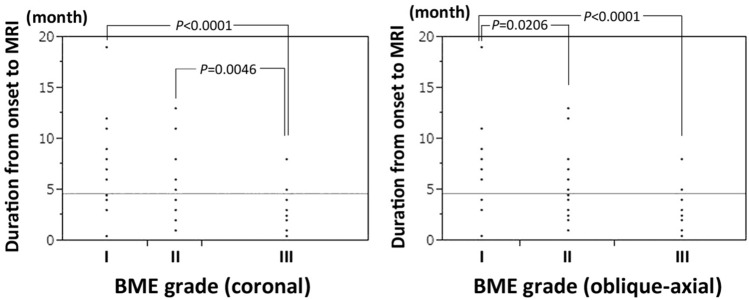
Relationship between the degree of extension of BME (BME grade) and the duration from the onset of hip pain to MRI examination. Duration from the onset of hip pain to MRI examination in the grade III were significantly shorter than grade I on both coronal and oblique-axial planes.

Results of univariate analysis for comparing the difference and non-difference groups according to the boundaries of necrotic lesions between T1- and CET1-weighted MR images are shown in Tables 1 and 2. Significant differences were found in the duration from the onset of hip pain to MR imaging examination between the two groups on both coronal (*P* < 0.0001) and oblique-axial planes (*P* = 0.0064). The BME grade in the difference group was significantly higher than in the non-difference group on the coronal plane (*P* = 0.0058). There were significant differences between the BME grade and duration from the onset of hip pain to MR imaging examination on both coronal and oblique-axial planes (Fig. 7).

**Table 1:** Univariate and multivariate analyses for coronal plane in the difference in the boundary of necrotic lesion group and the non-difference in the boundary of necrotic lesion group, comparing unenhanced and contrast-enhanced T1-weighted MR images.

	Difference group (*N* = 28)	Non-difference group (*N* = 40)	Univariate	Multivariate	Adjusted
			*P* values	*P* values	odds ratio
Age [year] (range)	40.3 (13–69)	35.5 (14–76)	0.0788	0.5762	
Sex	Male: 11 Female: 17	Male: 25 Female: 15	0.0591	–	
BMI [kg/m^2^] (range)	21.5 (16.9–29.7)	23.1 (17.6–32.5)	0.0947	–	
**Associated factor (%)**			0.2788	–	
Corticosteroid	18 (64)	19 (47)			
Alcohol	10 (36)	17 (43)			
Corticosteroid and alcohol	0 (0)	2 (5)			
Idiopathic	0 (0)	2 (5)			
ARCO stage (%)	IIIA: 21 (75) IIIB: 7 (25)	IIIA: 32 (80) IIIB: 8 (20)	0.6246	–	
JIC type (%)	B: 1 (4) C1: 10 (36) C2: 17 (60)	B: 3 (7) C1: 17 (43) C2:20 (50)	0.6155	–	
Demarcating sclerosis (%)	17 (61)	31 (78)	0.1349	–	
Onset to MRI [month] (range)	2.3 (0.5–8)	6.3 (1–19)	< 0.0001*	0.0008*	0.41
BME grade (%)	I: 2 (7) II: 6 (21) III: 20 (72)	I: 17 (42) II: 6 (16) III: 17 (42)	0.0058*	0.0811	

*BMI* body mass index, *MRI* magnetic resonance imaging, *ARCO* association research circulation osseous, *JIC* Japanese investigation committee, *BME* bone marrow edema.

*P < 0.05 indicates significance.

**Table 2:** Univariate and multivariate analyses for oblique-axial plane in the difference in the boundary of necrotic lesion group and the non-difference in the boundary of necrotic lesion group, comparing unenhanced and contrast-enhanced T1-weighted MR images.

	Difference group (*N* = 32)	Non-difference group (*N* = 35)	Univariate	Multivariate	Adjusted
			*P* values	*P* values	odds ratio
Age [year] (range)	37.9 (13–69)	36.0 (14–76)	0.5624	–	
Sex	Male: 15 Female: 17	Male: 14 Female: 21	0.5705	–	
BMI [kg/m^2^] (range)	21.8 (16.9–29.7)	22.9 (17.6–32.5)	0.2071	–	
**Associated factor (%)**			0.4018	–	
Corticosteroid	14 (44)	15 (43)			
Alcohol	18 (56)	17 (48)			
Corticosteroid and alcohol	0 (0)	2 (6)			
Idiopathic	0 (0)	1 (3)			
ARCO stage (%)	IIIA: 25 (78) IIIB: 7 (22)	IIIA: 27 (77) IIIB: 8 (23)	0.9233	–	
JIC type (%)	B: 2 (6) C1: 14 (44) C2: 16 (50)	B: 2 (6) C1: 16 (46) C2: 17 (48)	0.9854	–	
Demarcating sclerosis (%)	24 (75)	25 (71)	0.7418	–	
Onset to MRI [month] (range)	3.4 (0.5–9)	5.8 (0.5–19)	0.0064*	0.0143*	0.80
BME grade (%)	I: 5 (16) II 10 (31) III 17 (53)	I: 12 (34) II 11 (32) III 12 (34)	0.1600	–	

*P < 0.05 indicates significance.

**Figure 8:**
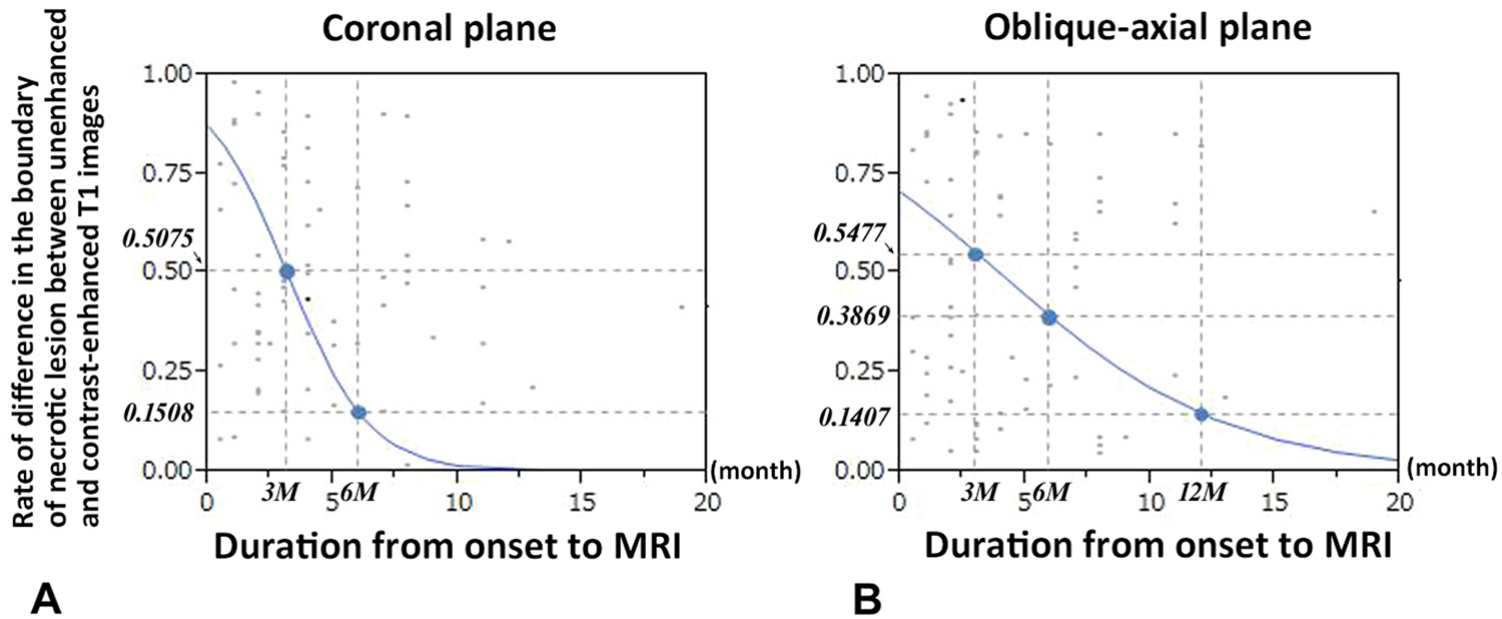
Simple logistic regression analyses for the relationship between the rate of presence of discrepancy in the definition of the boundary of necrotic lesion between unenhanced and contrast-enhanced T1 images and the duration from the onset of hip pain to MR imaging examination. (**a**) Results of the mid coronal slice. The rates of discrepancy 3 months (M) and 6 months after the onset are 0.5075 and 0.1508, respectively. (**b**) Results of mid oblique-axial slice. The rates of discrepancy 3, 6, and 12 months after the onset are 0.5477, 0.3869, and 0.1407, respectively.

Multivariate analyses revealed that the duration from the onset of hip pain to MR imaging examination in both coronal (*P* = 0.0008) and oblique-axial slices (*P* = 0.0143) were independently associated with differences in the boundaries of the necrotic lesions between unenhanced and CET1-weighted MR images (Tables 1 and 2). Simple logistic regression analysis revealed that unenhanced T1 images may be insufficient for the precise evaluation of the boundaries between necrotic and living bone in approximately half of ONFH cases 3 months after the onset of hip pain on both coronal and oblique axial planes (Fig. 8).

On the coronal FST2-weighed MR images, twenty-seven hips (40%) showed the differences in the boundaries of necrotic lesions compared with those of the CET1-weighted MR images. Significant differences were found in the sex (difference group vs. non-difference group; male 37% vs. male 63%, *P* = 0.0330), BMI (21.3 kg/m^2^ vs. 23.2 kg/m^2^, *P* = 0.0341), and duration from the onset of hip pain to MR imaging examination between the difference (3.0 months vs. 5.7 months, *P* = 0.0022) between the two groups. Multivariate analyses revealed that the sex (*P* = 0.0206) and duration from the onset of hip pain to MR imaging examination (*P* = 0.0434) in the coronal slice were independently associated with differences in the boundaries of the necrotic lesions between FST2- and CET1-weighted MR images.

Regarding the assessment of the boundaries of the necrotic lesion, intraobserver variabilities of observer 1 and 2 were 0.9096 and 0.8354 on the coronal slices, and 0.9054 and 0.8756 on the oblique-axial slices, which corresponded to an almost perfect score. Interobserver variabilities of the first and second assessments were 0.7661 and 0.7807 on the coronal planes, and 0.7740 and 0.7859 on the oblique-axial planes, which corresponded to a substantial agreement. Regarding the assessment of the degree of extension of BME, intraobserver variabilities of the observer 1 and 2 were 0.9236 and 0.9054 on the coronal slices, and 0.9154 and 0.8956 on the oblique-axial slices, which corresponded to an almost perfect score. Interobserver variabilities of the first and second assessments were 0.7940 and 0.8259 on the coronal planes, and 0.8661 and 0.8807 on the oblique-axial planes, which corresponded to an almost perfect or substantial agreement.

## Discussion

The T1 low-intensity band on magnetic resonance MR images is generally used to evaluate the boundary of the necrotic lesion as well as diagnose ONFH^16,17^. However, a previous study reported that the rates of detection of the low-intensity band were different between pre-collapse stage (ARCO stage I 100%, II 82%) and post-collapse stage (ARCO stage III 24%, IV 0%) based on unenhanced coronal images of MRI^18^. In contrast, the necrotic boundary was detected clearly in any stage based on CE coronal images of MRI of the hips^18^. Our recent study showed that BME on MR imaging was the most sensitive sign of the subchondral collapse, even though the femoral head was spherical on plan radiograph^23^. Therefore, we hypothesized that the necrotic boundary would be obscured by the BME secondary to collapse of the femoral head (T1 low-intensity band) on unenhanced MRI but would be clearly detected on CE MRI. In the current study, the boundaries of the necrotic lesion were clearly detected in almost all hips on the CE T1-images of MRI (coronal: 100%, oblique-axial: 97%). The BME grade in the group with difference of necrotic boundaries between unenhanced and CE T1 images, was significantly higher than the group without difference of necrotic boundaries between the two images.

Koo et al. reported that ONFH with BME at the initial MR examination (3 months after the onset of hip pain) had joint effusion and exhibited intense radionuclide uptake in the proximal femur^24^. This result corresponded to the extent of edema on MR images, and the BME resolved on follow-up MR images^23^. Meier et al. reported that the size of BME of the femoral head in ARCO stage III (early collapse stage) is larger than that in ARCO stage IV (late collapse stage)^25^. In this study, multivariate analyses revealed that the duration from the onset of hip pain to MR imaging examination in both coronal and oblique-axial slices, were independently associated with differences in the boundaries of necrotic lesions between T1- or FST2- and CET1-weighted MR images. In addition, the BME grade was significantly associated with the duration from the onset of hip pain to MRI examination. These findings were consistent with previous reports. According to the findings of the current study, approximately half of ONFH cases 3 months after the onset of hip pain had necrotic lesions with undefined boundaries on unenhanced T1 images of MRI (Fig. 8), suggesting that the use of unenhanced MR images may be insufficient for precise assessment of the boundaries of necrotic lesions for ONFH cases in the early phase of subchondral collapse due to the diffuse BME.

A previous study demonstrated that collapse inevitably occurs when the necrotic lesion extends laterally to the acetabular edge, suggesting the importance of the lateral boundaries of the necrotic lesions to the weight-bearing portion of the acetabulum upon collapse^1^. Nishii et al. reported that hips with less than 2 mm of collapse and necrotic lesions that were occupying less than the medial 2⁄3 of the weight-bearing area (Type A and B in Fig. 1) had a high chance of cessation of collapse and improvement of symptoms with no surgical intervention^10^. Our recent study showed that the collapse is more likely to occur in ONFH patients in which the boundary of a necrotic lesion is located more anteriorly, even in ONFH patients with medially located necrotic lesions^12^. Kubo et al. revealed that the cutoff point of anterior necrotic angle is 79° on occurrence of collapse^12^. Therefore, we consider that the precise evaluation of the boundary of necrotic lesion using CE-MR imaging is necessary to predict the joint prognosis, if the boundaries of necrotic lesions are unclear based on unenhanced T1- or FST2-weighted MR imaging.

With regard to the boundaries of necrotic lesions, among the cases with differences between T1- and CET1-weighted MR images, nine of 28 hips (32%) on the mid coronal and eight of 32 hips (25%) on the mid oblique-axial CET1 images were wider than those on the T1 images. Sugioka described that transtrochanteric anterior rotational osteotomy is indicated when one-third or more of the posterior intact articular surface to the femoral head remains intact^13^. Previous studies clearly demonstrated that 34% or more of postoperative intact ratio is necessary to prevent progressive collapse of the femoral head or the cut off value of postoperative intact ratio for preventing both progressive collapse and osteoarthritic change is 39.2% after transtrochanteric rotational osteotomy. These findings indicate that a little difference in postoperative intact articular surface to the femoral head may affect clinical and radiological results^26,27^. In our institution, approximately 10% of ONFH patients (55 of 588 patients, Fig. 2) were underwent CE MRI for the reason that it was difficult to assess the boundary of necrotic lesion using unenhanced MR image. Since underestimation of the boundaries of the necrotic lesions preoperatively leads to poor clinical and radiological results after the osteotomies, young patients with ONFH preferred joint-preserving procedures in which the boundaries of necrotic lesions is difficult to detect based on unenhanced T1- or FST2-weighted MR imaging, and in which CE MR imaging would be considered when the renal function is within the normal range.

The current study has several major limitations. First, histopathologic correlation with CE MR imaging was not confirmed in all hips, since only 26 of 72 cases (36%) underwent prosthetic replacement. Second, the true rate of difference in the boundary of necrotic lesion between unenhanced and CE T1 image is unclear, although the ratio of the difference group was high (coronal: 42%, oblique-axial: 48%) because CE MRI was performed only for patients with ONFH, in which it was difficult to assess the boundary of necrotic lesion using unenhanced MR images. Third, the oblique-axial plane (parallel to the femoral neck axis) of the MR image is uncommonly used for routine examination for the diagnosis of ONFH because this plane requires additional time for bilateral assessment comparing the axial plane parallel to the body axis. However, the rotation is made based on the femoral neck axis in transtrochanteric rotational osteotomy. Thus, the oblique-axial plane is necessary for determining the indication for transtrochanteric rotational osteotomy^28^. Fourth, comparison of the boundaries of the necrotic lesions between FST2- and CET1-weigthed MR images were made only with the coronal plane, since the oblique-axial FST2-weighted MR images are not routinely performed. However, the rate of difference of the boundary of necrotic lesions between coronal FST2- and CET1-weigthed MR images (40%) was similar with that between unenhanced T1- and CET1-weight images (42%). Finally, follow-up MR imaging was not performed in the same patients, as 70 of 72 cases (97%) underwent surgical treatments.

In conclusion, the BME on MRI may obscure the boundaries of necrotic lesions on unenhanced MR image in ONFH cases in the early phase of subchondral collapse and CE MRI can solve this problem.

## Data Availability

The datasets used and/or analyzed during the current study are available from the corresponding author on reasonable request.

## Abbreviations

AP: Anteroposterior
ARCO: Association research circulation osseous
BMI: Body mass index
CE: Contrast-enhanced
FS: Fat-saturated
MRI: Magnetic resonance imaging
ONFH: Osteonecrosis of the femoral head
